# Tamoxifen pharmacokinetics and pharmacodynamics in older patients with non-metastatic breast cancer

**DOI:** 10.1007/s10549-023-06925-z

**Published:** 2023-04-17

**Authors:** E. T. D. Souwer, A. Sanchez-Spitman, D. J. A. R. Moes, H. Gelderblom, J. J. Swen, J. E. A. Portielje, H. J. Guchelaar, T. van Gelder

**Affiliations:** 1 Department of Medical Oncology, Leiden University Medical Center, Albinusdreef 2, P.O. Box 9600, 2300 RC Leiden, The Netherlands; 2 Department of Clinical Pharmacy and Toxicology, Leiden University Medical Center, Leiden, The Netherlands

**Keywords:** Breast cancer, Adjuvant treatment, Tamoxifen, Therapeutic drug monitoring, Pharmacokinetics, Pharmacodynamic

## Abstract

**Background:**

We aimed to study the pharmacokinetics and -dynamics of tamoxifen in older women with non-metastatic breast cancer.

**Methods:**

Data for this analysis were derived from the CYPTAM study (NTR1509) database. Patients were stratified by age (age groups < 65 and 65 and older). Steady-state trough concentrations were measured of tamoxifen, N-desmethyltamoxifen, 4-hydroxy-tamoxifen, and endoxifen. *CYP2D6* and *CYP3A4* phenotypes were assessed for all patients by genotyping. Multiple linear regression models were used to analyze tamoxifen and endoxifen variability. Outcome data included recurrence-free survival at time of tamoxifen discontinuation (RFSt) and overall survival (OS).

**Results:**

668 patients were included, 141 (21%) were 65 and older. Demographics and treatment duration were similar across age groups. Older patients had significantly higher concentrations of tamoxifen 129.4 ng/ml (SD 53.7) versus 112.2 ng/ml (SD 42.0) and endoxifen 12.1 ng/ml (SD 6.6) versus 10.7 ng/ml (SD 5.7, *p* all < 0.05), independently of *CYP2D6* and *CYP3A4* gene polymorphisms. Age independently explained 5% of the variability of tamoxifen (b = 0.95, *p* < 0.001, *R*^2^ = 0.051) and 0.1% of the variability in endoxifen concentrations (b = 0.45, *p* = 0.12, *R*^2^ = 0.007). Older patients had worse RFSt (5.8 versus 7.3 years, *p* = 0.01) and worse OS (7.8 years versus 8.7 years, *p* = 0.01). This was not related to differences in endoxifen concentration (HR 1.0, 95% CI 0.96–1.04, *p* = 0.84) or *CYP* polymorphisms.

**Conclusion:**

Serum concentrations of tamoxifen and its demethylated metabolites are higher in older patients, independent of *CYP2D6* or *CYP3A4* gene polymorphisms. A higher bioavailability of tamoxifen in older patients may explain the observed differences. However, clinical relevance of these findings is limited and should not lead to a different tamoxifen dose in older patients.

## Background

The number of older women diagnosed with breast cancer is rising, and more than 40% of these are above 65 years of age [1]. Around 80% of newly diagnosed older patients have hormone receptor-positive breast cancer [2]. In early-stage hormone receptor-positive breast cancer, tamoxifen is still a major option for adjuvant endocrine treatment [3].

Therapeutic drug monitoring (TDM) in patients with breast cancer receiving tamoxifen is no standard practice and its added value is under debate [4]. Tamoxifen pharmacokinetics is influenced by cytochrome P450 genetic polymorphisms. Polymorphisms in the *CYP2D6* gene, and to a lesser degree in the *CYP3A4* gene, influence tamoxifen metabolism resulting in variation in tamoxifen metabolites, of which 4-hydroxy-N-desmethyltamoxifen (endoxifen) has the highest affinity for the estrogen receptor. *CYP2D6* poor metabolizers are at risk for undertreatment because of subtherapeutic levels of endoxifen and precursors; however, the impact of *CYP2D6* on oncological outcomes such as recurrence risk and overall survival is unclear [5]. Although a possible correlation exists between tamoxifen metabolite concentrations and treatment-related toxicity[6, 7], tamoxifen dosage corrected for *CYP2D6* genotype did not show a significant increase in treatment-related toxicity [8, 9]. Previous smaller studies showed an increase in tamoxifen and endoxifen concentrations with age during steady-state treatment [10, 11] independent of *CYP2D6* and *CYP3A4* genotype and phenotype [12, 13]. Age-related changes in hepatic metabolism of drugs have also been found for other drugs, including tacrolimus, for which after normalization for dose and body weight the trough concentrations were more than 50% higher in older adults than young adults [14].

Selecting older patients for adjuvant breast cancer treatment is challenging as treatment benefit can be diminished by shorter life expectancies due to competing risk for survival. In addition, older patients are more likely to discontinue adjuvant treatment [15], limiting the benefit of tamoxifen as adjuvant treatment.

We aimed to compare the pharmacokinetics and -dynamics of tamoxifen in older and younger women with non-metastatic breast cancer.

## Patients and methods

Data for this analysis were derived from the CYPTAM study (NTR1509) database. More detailed information on the CYPTAM study was published previously [8, 16–22]. In short, from February 2008 till December 2010, patients with early breast cancer receiving adjuvant tamoxifen (20 mg QD) were recruited in the multicenter prospective CYPTAM study in The Netherlands and Belgium. All patients gave written informed consent.

After inclusion in the CYPTAM study and having used tamoxifen for more than 2 months but less than 1 year, serum samples were collected for measurement of tamoxifen (and metabolite) concentrations and whole blood for *CYP2D6* and *CYP3A4* genotyping.

At enrollment, clinical information was retrospectively collected and registered. Demographics, tumor, and treatment information including duration of adjuvant therapy and outcome data were recorded by professional data managers. Premenopausal and postmenopausal information was not available. For information concerning relapse-free survival (RFS) and overall survival, last month of follow-up was between December 2016 and February 2017. Data were censored after this date, or at last-know follow-up for RFS. Relapse-free survival (RFS) at the time of tamoxifen discontinuation (RFSt) was also used as endpoint to avoid effect modification by subsequent aromatase inhibitor use.

Trough levels were obtained at least 12 h after the last intake of tamoxifen. Steady-state concentrations of tamoxifen and its metabolites (N-desmethyltamoxifen, 4-hydroxy-tamoxifen, and endoxifen) were measured in serum with high-performance liquid chromatography-tandem mass spectrometry (HPLC/MS/MS) as described earlier [23] and metabolic ratios were calculated for tamoxifen-N-desmethyltamoxifen, tamoxifen-4-hydroxy-tamoxifen, N-desmethyltamoxifen / endoxifen, and 4-hydroxy-tamoxifen-endoxifen. An endoxifen concentration < 5.9 ng/ml was considered subtherapeutic [24]. In addition to this threshold, other suggested thresholds of 9 nM [25] and 14 nM for endoxifen [26] and 3.26 for 4-hydroxy-tamoxifen [27]) were investigated.

*CYP2D6* Genotyping was performed with Amplichip CYP450 test (Roche Diagnostic, Indianapolis, USA). In accordance with their *CYP2D6* genotypes, all individuals were classified in predicted phenotypes: ultra-rapid metabolizer (UM), normal metabolizer (NM), intermediate metabolizer (IM), and poor metabolizer (PM).

The considered CYP2D6-predicted phenotypes were defined as follows: ultra-rapid (duplication of fully active alleles), normal (with two fully active alleles), intermediate (one fully active allele and one nonactive allele, two low activity alleles or a combination of one low activity allele and one inactive allele), and poor metabolizers (with two inactive alleles). Alleles with decreased CYP2D6 activity were *9, *10, *17, *29, *36, *41, *10xN, *17xN, and *41xN, whereas *CYP2D6* inactive alleles were *3, *4, *5, *6, *7, *8, *11, *14A, *15, *19, *20, *40, and *4xN [28].

CYP3A4*22 was analyzed with TaqMan 7500 (Applied Biosystems, Nieuwerkerk a.d. IJssel, The Netherlands) with predesigned assays, according to the manufacturers’ protocol.

### Statistical analysis

For this study, patients were stratified by age (age groups < 65 and 65 and older). Baseline characteristics were reported as means with standard deviation (SD) or as frequencies and percentages.

Pharmacokinetic and pharmacodynamic outcomes were compared using linear regression and T-test to compare continuous variables and chi-square or.analysis of variance (ANOVA) for differences between age and patient groups including four phenotypes (PM, IM, NM, and UM). To assess the variability in concentrations of tamoxifen and endoxifen for age and *CYP2D6* phenotype, linear multivariable regression models were constructed. Age was investigated as categorical and as a continuous independent variable in the linear multivariable regression models. R-Squared (*R*^2^) was used to determine the proportion of variability that could be explained by the independent variables. For overall survival analyses, a cox-proportional hazard model was constructed. Independent variables included age, histology grade, tumor and nodal status, endoxifen concentration, and *CYP2D6* and *CYP3A4* phenotypes. All analyses were performed using SPSS version 23.0 (SPSS, Inc., Chicago, IL). *p*-values less than 0.05 were considered statistically significant.

## Results

All 667 patients from the CYPTAM database were included; from one patient age was missing. There were less than 2% missing demographic characteristics, and for 59 patients (9%), no metabolic ratios could be assessed. *CYP2D6* en *CYP3A4* genotypes were not available in 29 (4%) and 30 (4%) patients, respectively.

At enrollment, median tamoxifen therapy duration was 0.37 year (range 0.23 to 0.60 years). Median follow-up was 6.4 years (range 0.1 to 9.3 years), and for RFSt and OS, 99 and 69 events were recorded, respectively.

Baseline characteristics are presented in Table 1. Mean age was 56.4 years, 141 (21%) were older than 65 years, *CYP2D6* phenotype and *CYP3A4* genotype were equally distributed among age groups, and 11% of the younger patients and 7% of older patients were CYP3A4*22 carrier (*p* = 0.34).

**Table 1:** Baseline characteristics

Patient characteristics	Missing *n*	Total *n*		<65 years		≥65 years		*p* value*
Total *n*		667		526		141		
Age, years (mean, SD)		56.4	(11.1)	52.0	(7.5)	72.6	(5.8)	<0.001
Age categories								
23–65		526	(78.9)					
65–75		102	(15.3)					
75+		39	(5.8)					
CYP2D6-metabolizer	29							
UM		5	(0.7)	5	(1.0)	0		0.11
NM		317		240	(45.6)	77	( 54.6)	
IM		269		223	(42.4)	46	(32.6)	
PM		47	(7.0)	38	(7.2)	9	(6.4)	
CYP3A4*22	30							0.34
*1/*22		73	(10.9)	58	(11.0)	10	(7.1)	
*22/*22		1	(0.1)	1		0		
Tumour stage	9							0.62
T1		356	(53.4)	278	(52.9)	78	(55.3)	
T2		274	(41.1)	218	(41.4)	56	(39.7)	
T3/4		28	(4.2)	24	(4.6)	4	(2.8)	
Nodal stage	3							0.06
0		317	(47.5)	237	(45.1)	80	(56.7)	
1		266	(39.9)	215	(40.9)	51	(36.2)	
2		57	(8.5)	50	(9.5)	7	(5.0)	
3		24	(3.6)	22	(4.2)	2	(1.4)	
PR	10							0.14
Positive		530	(79.5)	426	(81.0)	104	(73.8)	
Negative		127	(19.0)	92	(17.5)	35	(24.8)	
HER2	3							0.19
Negative (0/1+)		574	(86.1)	448	(85.2)	126	(89.4)	
Positive (2+/3+)		90	(13.5)	76	(14.4)	14	(9.9)	
Grade	7							0.91
1		94	(14.1)	72	(13.7)	22	(15.6)	
2		378	(56.7)	300	(57.0)	78	(55.3)	
3		188	(28.2)	148	(28.1)	40	(28.4)	
Type of surgery	5							0.02
Mastectomie		310	(46.5)	231	(43.9)	79	(56.0)	
Breast conserving		352	(52.8)	292	(55.5)	60	(42.6)	
Adjuvant radiotherapy	3	462	(69.3)	383	(72.8)	79	(56.0)	0.001
Adjuvant chemotherapy	3	407	(61.0)	390	(74.1)	17	(12.1)	<0.001
Tamoxifen (years, mean, SD)	30	2.9	1.5	3.0	1.5	2.7	1.3	0.1

Mean with standard deviation (SD) and frequencies with percentage (%) * between groups < 65 and > = 65

There was a non-significant difference in nodal stage and type of surgery; patients ≥ 65 had less nodal involvement (N0; 57% versus 45%, *p* = 0.06) and a higher number of older patients underwent a mastectomy compared to their younger counterparts. Older patients were less likely to receive adjuvant radiotherapy (56% versus 73%) and chemotherapy (12% versus 74%). Between age groups, there was no difference in tumor stage, tumor grade, or the duration of adjuvant tamoxifen treatment (mean 2.7 and 3.0 years, *p* = 0.96). A total of 424 patients (64%) switched to an Aromatase Inhibitor (AI) as treatment strategy; other reasons were side effects (*n* = 56, 8%), disease recurrence (*n* = 37, 6%), or other (*n* = 112, 17%).

Pharmacokinetic information stratified by age group is presented in Table 2. Older patients were more likely to have higher serum concentrations of tamoxifen and its metabolites: tamoxifen 129.4 ng/ml (SD 53.7) versus 112.2 ng/ml (SD 42.0), endoxifen 12.1 ng/ml (SD 6.6) compared to 10.7 ng/ml (SD 5.7), *p* < 0.05. Metabolic ratios were similar among age groups.

**Table 2:** Pharmacokinetics stratified by age group

	< 65 years	≥ 65 years	*p*-value
Tamoxifen (ng/ml)	112.2 (42.0)	129.4 (53.7)	0.001
N-desmethyltamoxifen (ng/ml)	210.5 (77.4)	233.0 (88.4)	0.001
4-Hydroxytamoxifen (ng/ml)	1.9 (0.8)	2.3 (1.1)	< 0.001
Endoxifen (ng/ml)	10.7 (5.7)	12.1 (6.6)	0.02
MR Tamoxifen-N-Desmethyltamoxifen	0.5 (0.1)	0.5 (0.1)	0.28
MR tamoxifen-4-hydroxy tamoxifen	64.8 (25.5)	63.7 (28.5)	0.66
MR N-desmethyltamoxifen-endoxifen	28.6 (25.1)	30.1 (29.7)	0.58
MR 4-hydroxy-tamoxifen-endoxifen	0.2 (0.8)	0.2 (0.1)	0.15
Number of patient endoxifen < 5.9 ng/ml (%)	110.0 (21.2)	27.0 (19.1)	0.58

Mean with standard deviation (SD) or frequency with percentage (%)

*MR* metabolic ratio

*CYP2D6* PM had lower concentrations of endoxifen and precursor metabolites, also reflected in a difference in metabolic ratio, although metabolic ratios were similar across age groups (Table 2). Pharmacokinetics stratified by *CYP2D6* phenotype and age group can be found in Appendix A and B, respectively. Linear regression analysis to assess the independent effect of age on variability in tamoxifen and endoxifen concentrations showed that age independently explained up to 5.1% of the variability, independently of *CYP2D6* phenotype (tamoxifen b = 0.94, *p* < 0.001, *R*^2=^0.05, endoxifen b = 0.05, *p* = 0.01, *R*^2^ = 0.007). Also, when *CYP3A4* genotype was included in the model, age still independently explained 5.2% of the variability of tamoxifen (b = 0.92, *p* < 0.001, *R*^2^ = 0.052) and 1% of the variability in endoxifen concentrations (b = 0.05, *p* < 0.01, *R*^2^ = 0.01). Seventeen (5%) NMs, 67 (25%) IMs, and 44 (94%) PMs had subtherapeutic endoxifen concentrations (< 5.9 ng/ml). The number of patients with endoxifen concentrations < 5.9nmg/ml was similar across age groups; 22% versus 19%, *p* = 0.54. In a small subgroup of *CYP3A4* heterozygous *1/*22 and homozygous *22/*22 patients (*n* = 74), age was no longer related to the endoxifen concentration (*p* = 0.59).

The proportion of patients that discontinued treatment due to side effects was similar across age groups (10% versus 9%, *p* = 0.57). There was a significant difference in mean RFSt and OS between older and the younger age groups; 5.75 versus 7.28 years (*p* = 0.04) and 7.79 versus 8.68 years (*p* = 0.01), respectively, Table 3. Median overall survival was not reached for both groups at the end of follow-up. There was no association between RFSt and *CYP2D6* (*p* = 0.39), age (*p* = 0.43) or endoxifen concentration (*p* = 0.21). In a multivariate model for overall survival, age, histology grade, and nodal stage significantly impacted the outcome; however, endoxifen concentration did not (HR 1.0, 95% CI 0.96–1.04, *p* = 0.84, Table 4). This did not change when threshold endoxifen concentration < 5.9 ng/ml (yes or no) instead of endoxifen concentrations as a continuous variable were included in the model (HR 1.13, 95% 0.96–1.04, *p* = 0.70).

**Table 3:** Pharmacodynamic outcome stratified by age group

Patient characteristics	< 65 years	≥ 65 years	*p*-value
	525	141	
Number of discontinuation (%)	42 (8)	14 (10)	0.57
RFSt (years)	7.28 (6.96–7.60)	5.75 (4.93–6.56)	0.01
OS (year)	8.68 (8.50–8.80)	7.79 (7.43–8.16)	0.04

Mean with standard deviation (SD) and frequencies with percentage (%)

*RFSt* relapse-free survival at time of tamoxifen discontinuation, *OS* overall survival

**Table 4:** Pharmacodynamic outcome model overall survival

Patient characteristics	HR with 95% CI	*p*-value
Age	(1.01–1.08)	0.03
Tumor stage	(0.88–1.32)	0.46
Histology grade	(1.02–1.52)	0.03
Nodal Stage	(1.10–1.93)	0.01
Endoxifen concentration	(0.96–1.04)	0.84

Hazard Ratio (HR) with 95% Confidence Interval (CI)

We could also not demonstrate an association between other thresholds of endoxifen (9 nM or 14 nM) or of 4-hydroxy tamoxifen below 3.26 nM and outcomes (RFSt or OS, data not shown).

## Discussion

This study set out to further investigate the pharmacokinetics and pharmacodynamics of tamoxifen in older women with non-metastatic breast cancer. We showed that age has a small statistically significant, but most unlikely a clinically relevant impact on the pharmacokinetics of tamoxifen. In older women, higher concentrations of endoxifen and precursors were found, independent of *CYP2D6* and *CYP3A4* phenotypes. Despite higher concentrations of endoxifen, discontinuation rates were not higher in older women and there was no effect on relapse rates among patients with levels of endoxifen which have previously been classified as subtherapeutic [24].

That age has a small but significant effect on endoxifen concentrations, independent of *CYP2D6* polymorphism and *CYP2D6* inhibitors, and was earlier reported by Teft et al. in 196 tamoxifen-treated patients [12] and by Lien et al. in a cohort of 151 patients [13]. In both studies, and in line with our study, the effect of age was small. Older studies that did not assess *CYP2D6*-polymorphisms showed similar trends [10, 11]. In contrast, a more recent study by Puszkiel found lower concentrations of endoxifen in older patients, but a smaller proportion of patients was aged ≥ 65 years (5.6%) in their study [29]. An alternative explanation for the higher concentrations [30] may be menopausal state resulting in higher bioavailability of tamoxifen, although this mechanism is still poorly understood.

In the CYPTAM study, we could not demonstrate an effect of low concentrations of endoxifen or *CYP2D6* genotype on recurrence-free survival or overall survival, as previously reported [18]. This is in line with the study of Hertz et al. that included 817 adjuvant treated breast cancer patients [31] and the study by Rae et al. [32]. *CYP2D6*-based dosing of tamoxifen was studied in 186 metastatic patients and did not show a difference in 6 months of PFS [33], questioning the clinical relevance of therapeutic drug monitoring (TDM) of tamoxifen. These negative associations are in contrast with two other large observational studies that reported that low levels of endoxifen [25] or *CYP2D6* PM and UM were associated with worse prognosis [6].

The difference in relapse-free and overall survival between age groups in our study is most likely caused by the fact that elderly received less adjuvant treatment or competing risk of death.

Previously, several explanations for the higher endoxifen concentrations in older patients have been postulated. With increasing age, *CYP3A4* activity may decrease. *CYP3A4* *22 carriers have reduced *CYP3A4* activity and thereby a higher first-pass effect in the liver, resulting in higher tamoxifen bioavailability and higher concentrations of tamoxifen metabolites [12]. However, in our study, the number of *CYP2D6* *22 was similar across age groups, and additionally, we showed that the higher concentrations of endoxifen were independent of *CYP3A4* genotype. This is supported by the lack of difference in metabolic ratios across age groups. Hence, most endoxifen is formed from N-desmethyl-tamoxifen, also in older patients, suggesting a difference in bioavailability instead of CYP450 activity. Alternative explanations include a reduced *CYP3A4* activity in the larger intestine caused by, for example, low vitamin D even in non-*CYP3A4* *22 carriers [12]. In this study, information regarding comedication was unavailable; hence, the number of patients with endoxifen levels below 5.9 ng/ml was not higher in older patients, thereby suggesting a limited effect of comedication in our population.

A possible selection bias and the limitation of a limited follow-up also applied to this study. The prevalence of *CYP2D6* PM was 7.1%, like other studies (5.2%-8.6%) [6, 13, 32]. By measuring just one serum sample, we cannot exclude that systemic endoxifen exposure changes over time. However, intra-patient variability for endoxifen has been shown to be low (0–19%) [34–36]. The availability of a limited number of samples also reflects clinical practice. Also, the lack of information on comorbidity and comedication could be regarded a limitation, although the effect of comedication in this study was deemed low. Due to the low number of events, and relative short follow-up, median OS could not be reached. The CYPTAM study may have been underpowered to detect the clinical outcome differences, affecting the generalizability of our results.

Deciding on adjuvant treatment for all patients requires patient tailored information on the absolute benefit of treatment, considering risk factors that strongly affect treatment outcome. For decisions on adjuvant endocrine treatment, prediction tools are available, such as PREDICT tool [37].

More recently, it was shown that individual risk estimation for older patients could be improved by incorporating comorbidity and functional parameters, considering the individual competing risk of death [38]. Given the complexity of tamoxifen metabolism, in order to improve the prediction of tamoxifen efficacy and safety, large studies with complex analysis would be required [39]. With current knowledge, TDM of tamoxifen in older patients is therefore even less likely to impact the outcome.

## Conclusions

Serum concentrations of tamoxifen and its demethylated metabolites including endoxifen are higher in older women on steady-state adjuvant tamoxifen treatment compared to younger women. The effect of age is independent of *CYP2D6* or *CYP3A4* gene polymorphisms. A higher bioavailability of tamoxifen in older patients may explain the observed differences. Treatment outcomes were worse in older patients, but this is partly due to tumor characteristics, less adjuvant treatment, or other cause mortality. These results do not support different tamoxifen dosing in older patients, nor do they support using TDM of tamoxifen in older patients.

## Data Availability

Enquiries about data availability should be directed to the authors.

## Appendix

See Tables 5, 6, 7, and 8.

**Table 5:** Pharmacokinetics by *CYP2D6* genotype

	PM	IM	NM	*p*-value
Tamoxifen (ng/ml)	117.6 (45.9)	118.9 (49.6)	111.9 (41.2)	0.18
N-desmethyltamoxifen (ng/ml)	277.4 (87.7)	232.6 (82.0)	190.7 (68.3)	< 0.001
4-Hydroxytamoxifen (ng/ml)	1.4 (0.5)	1.8 (0.8)	2.2 (0.9)	< 0.001
Endoxifen (ng/ml)	3.6 (1.7)	9.2 (4.9)	13.4 (5.4)	< 0.001
MR tamoxifen-N-Desmethyltamoxifen	0.4 (0.1)	0.5 (0.1)	0.6 (0.1)	< 0.001
MR tamoxifen-4-hydroxy tamoxifen	93.9 (39.2)	72.4 (26.5)	54.3 (16.9)	< 0.001
MR N-desmethyltamoxifen-endoxifen	90.9 (35.2)	32.1 (19.4)	17.1 (11.3)	< 0.001
MR 4-hydroxy tamoxifen-endoxifen	0.4 (0.1)	0.2 (0.1)	0.2 (0.04)	< 0.001

Mean with standard deviation (SD), *MR* metabolic ratio, *PM* poor metabolizer

^*^Other: *IM* intermediate metabolizer, *NM* normal metabolizer

**Table 6:** Pharmacokinetics stratified *CYP2D6* genotype

	PM	Other*	*p*-value
	*n* = 47	*n* = 590	
Endoxifen (ng/ml)	3.6 (1.7)	11.6 (5.6)	< 0.001
MR N-desmethyltamoxifen-endoxifen	90.9 (35.2)	23.9 (17.2)	< 0.001

Mean with standard deviation (SD), *MR* metabolic ratio, *PM* poor metabolizer

^*^Other: *IM* intermediate metabolizer, *NM* normal metabolizer

**Table 7:** Pharmacokinetics stratified *CYP2D6* genotype in < 65 years

	PM	Other*	*p*-value
	*n* = 38	*n* = 463	
Endoxifen (ng/ml)	3.6 (1.9)	11.3 (5.5)	< 0.001
MR N-desmethyltamoxifen-endoxifen	91.5 (37.5)	37.5 (6.2)	< 0.001

Mean with standard deviation (SD), *MR* metabolic ratio, *PM* poor metabolizer

*Other: *IM* intermediate metabolizer, *NM* normal metabolizer

**Table 8:** Pharmacokinetics stratified by *CYP2D6* genotype in > 65 years

	PM	Other*	*p*-value
	*n* = 9	*n* = 123	
Endoxifen (ng/ml)	3.6 (1.0)	12.1 (6.6)	< 0.001
MR N-desmethyltamoxifen-endoxifen	87.9 (21.4)	26.1 (25.1)	< 0.001

Mean with standard deviation (SD), *MR* metabolic ratio, *PM* poor metabolizer

*Other: *IM* intermediate metabolizer, *NM* normal metabolizer
